# Choice of Renal Function Estimator Influences Adverse Outcomes with Dabigatran Etexilate in Patients with Atrial Fibrillation

**DOI:** 10.1055/s-0038-1676356

**Published:** 2018-12-10

**Authors:** Bryan H. Simpson, David M. Reith, Natalie J. Medlicott, Alesha J. Smith

**Affiliations:** 1 School of Pharmacy, University of Otago, Dunedin, New Zealand; 2 Dunedin Medical School, University of Otago, Dunedin, New Zealand

**Keywords:** dabigatran etexilate, hemorrhage, cerebrovascular accident, systemic embolism, renal function

## Abstract

**Background** Clinical significance of dosing dabigatran with different estimates of renal function for treatment of atrial fibrillation (AF) is unknown. Renal function is routinely estimated by the chronic kidney disease epidemiology initiative equation (CKD-EPI) and used to guide dosing. The aim of this study was to investigate the risk of adverse outcomes for patients with AF when different estimators of renal function are used.

**Material and Methods** AF patient data were extracted from national administrative databases. Renal function was estimated using Cockcroft–Gault, CKD-EPI, and CKD-EPI adjusted for body surface area (CKD-EPI-BSA). Outcomes of cerebrovascular accident (CVA), systemic embolism (SE), and hemorrhage were extracted.

**Results** In total, 2,425 patients were identified, of which there were hospitalizations for 138 (5.7%) hemorrhagic events, 45 (1.9%) CVA/SE, and 33 (1.4%) unspecified CVA. The level of agreement between Cockcroft–Gault with CKD-EPI and CKD-EPI-BSA yielded a weighted kappa statistic of 0.47 and 0.71, respectively. CKD-EPI and CKD-EPI-BSA significantly overestimated renal function in elderly patients resulting in higher recommended doses compared with Cockcroft–Gault. The hazard ratio for a hemorrhagic event was 2.32 (95% confidence interval, 1.22–4.42; *p* = 0.01) when a high dose was given compared with normal dose, based on Cockcroft–Gault.

**Conclusion** Both CKD-EPI and CKD-EPI-BSA equations significantly overestimated renal function in the elderly population compared with the Cockcroft–Gault equation. This may lead to dose selection errors for dabigatran, particularly for those with severe impairment, increasing the risk of adverse outcome. Hence, CKD-EPI and CKD-EPI-BSA equations should not be substituted for the Cockcroft–Gault equation in the elderly for the purpose of renal dosage adjustments.

## Background

Previous studies have shown that using different estimators of renal function can result in different doses of dabigatran etexilate, a non–vitamin K antagonist oral anticoagulant (NOAC), being prescribed to patients. 1 2 3 4 5 6 Approximately 80% of dabigatran etexilate is eliminated by renal excretion, 7 making it important to ensure renal function is estimated appropriately when selecting individual doses. It has also been reported that there is a relationship between clinically inappropriate dosing of NOACs (including dabigatran etexilate) and adverse clinical outcomes. 8 9 Currently, there are no reported data showing how the use of different renal function estimators affects patient outcomes for individuals with atrial fibrillation (AF) who receive treatment with dabigatran etexilate.

The Cockcroft–Gault equation 10 has long been used in clinical care, since its development in 1976, to evaluate renal function by estimating creatinine clearance (CrCl). More recently, alternative methods have been adopted such as the original modification of diet in renal disease (MDRD) equation, the abbreviated MDRD equation (4) (MDRD-4), and the chronic kidney disease epidemiology initiative 11 (CKD-EPI). These latter equations provide estimates of glomerular filtration rate (eGFR) normalized to a body surface area (BSA) of mL/min/1.73 m ^2^ . It has been reported that CKD-EPI gives the best estimation of glomerular filtration rate of these three equations. 12 However, despite this, the recommended guidance for evaluation of pharmacokinetics of medicines in patients with decreased renal function from the United States Food and Drug Administration (FDA) 13 and European Medicines Agency (EMA) 14 does not specify one particular method of estimation. This allows for worldwide inconsistency in dosage recommendations in patients with decreased renal function.

In New Zealand, CKD-EPI is the renal function estimate of choice for clinical care and is now routinely reported in patient notes following plasma creatinine blood tests. 15 It is probable that CKD-EPI is being used to guide dabigatran etexilate dosing 16 despite clinical guidance from the sponsor and international scientific societies recommending the Cockcroft–Gault equation. 17 18 19 When eGFR is used by clinicians to guide dabigatran etexilate dosing, there is a need to adjust the result for the individual patient's BSA, especially at the extremes of body size. 20 Failing to adjust for BSA has the potential to lead to incorrect estimation of renal function and result in inappropriate dose selection. Currently, there is no guidance to adjust for those patients who have a BSA not close to 1.73 m ^2^ . Furthermore, the presence of different methods of estimating renal function has created some confusion for clinicians as to the best approach in clinical practice. 20 21

The aim of the present study was to investigate the difference between different estimators of renal function and the risk of adverse outcomes (hemorrhage or thromboembolism) for patients with AF when these different estimators are used for dose selection of dosing dabigatran etexilate. To achieve this, we have used a high-quality observational dataset encompassing several high volume centers to address this issue.

## Methods

### Identification of Study Cohort

This was a retrospective cohort study using administrative health data from New Zealand. The databases accessed were the Best Practice Intelligence (BPI) database operated by Best Practice Advocacy Centre Clinical Solutions, New Zealand, 22 and the New Zealand Ministry of Health Pharmaceutical Collection 23 (PC). The BPI database is a secure, internet-based, reporting tool that uses data downloaded from the enrolled general practice patient electronic health record (EHR) and covers about 20% of the New Zealand population. The PC contains prescription details about pharmaceutical dispensing claims for dabigatran etexilate along with other prescribed medicines as well as information on gender, date of birth, age, ethnicity, frequency, and quantity dispensed for all of the New Zealand population. The study population included patients: with a diagnosis of AF by the general practitioner (READ codes G573, G5730, G5731, G5732, G573z); aged 18 years or older; had at least one dispensing of dabigatran etexilate during the study period between July 1, 2011 (when dabigatran etexilate became available in New Zealand), and December 31, 2015; serum creatinine measurements within 60 days before or 30 days after their first dispensing of dabigatran etexilate; at least one height measurement; and bodyweight measurements within 1 year before or after their first dispensing of dabigatran etexilate. If multiple serum creatinine or bodyweight measurements were recorded, the measurement closest to the initiation of dabigatran etexilate initiation was used. Weight measurements more than five standard deviations from the mean were considered to be data entry errors and were excluded. If multiple height measurements were recorded for an individual patient, the mean was calculated, with any measurement more than two standard deviations from the mean height being excluded. Any patients who had a height measurement (either as a single or average measurement) that was more than five standard deviations from the cohort mean were excluded. Where an alternative weight or height measurement was not recorded, the patient was excluded from the cohort. The information from different datasets were linked using each patient's encrypted National Health Index number (NHI number; a life-long unique identifier for all interactions with the New Zealand health system) to ensure patient anonymity. Ethical approval was obtained from the University of Otago, New Zealand Ethics Committee (Reference: HD15/054).

### Patient Covariates

Dispensed medications, patient demographic, and covariate data were extracted from the PC and BPI databases for patients who meet the inclusion criteria. Patients were categorized into age groupings of under 65 years, 65 to 74 years, 75 to 79 years, and over 80 years to align to both regulatory and the categories used by the sponsor to guide dosing. 17 24 The treatment period with dabigatran etexilate was determined by the number of days supplied for each series of continuous treatment. Continuous dabigatran etexilate use was defined as one or more dispensings recorded in the PC with less than 120 days between dispensing (prescriptions in New Zealand for dabigatran etexilate typically supply 90 days which are dispensed in 30 day amounts). When 120 days or more elapsed between dabigatran etexilate prescriptions, a patient was considered to have ceased dabigatran etexilate treatment. If the patient restarted dabigatran etexilate treatment after 120 days or more had elapsed, they were considered as a new patient in the study.

#### Estimation of Renal Function

Baseline renal function was estimated via three different methods using the serum creatinine measurement closest to the first dispensing of dabigatran etexilate:

1. Cockcroft–Gault 10 equation using equation (1):

where:

CrCl = creatinine clearance,

age = age in years,

weight = weight in kg,

SCr = serum creatinine (expressed in mg/dL).

(1)

1. Chronic kidney disease epidemiology collaboration (CKD-EPI) 11 equation using equation (2):

where:

eGFR = estimated glomerular filtration rate,

κ = 0.7 for females and 0.9 for males,

α =  − 0.329 for females and −0.411 for males,

min indicates the minimum of SCr/κ or 1,

max indicates the maximum of SCr/κ or 1,

age = patient age in years

SCr = serum creatinine (expressed in mg/dL).

(2)

1. Chronic kidney disease epidemiology collaboration adjusted for BSA (CKD-EPI-BSA) equation using equation (3):

where:

eGFR = estimated glomerular filtration rate,

κ = 0.7 for females and 0.9 for males,

α =  − 0.329 for females and −0.411 for males,

BSA= individuals body surface area,

min indicates the minimum of SCr/κ or 1,

max indicates the maximum of SCr/κ or 1,

SCr = serum creatinine (expressed in mg/dL).

BSA = √[(height (cm) × weight (kg))/3,600]

(3)

Patients were classified per equation according to the renal impairment dose stratification for AF according to the sponsors medicines data sheet. 17 These were: (1) ≥50 mL/min—no dose adjustment required (i.e., 300 mg daily); (2) ≥30 mL/min and <50 mL/min—dose reduction to 220 mg daily; (3) <30 mL/min—use contraindicated.

### Patient Outcomes

The outcomes of interest were any admission to hospital for hemorrhage or thromboembolism ( Supplementary Table 1 ) and were extracted from the New Zealand Ministry of Health National Minimum Dataset (NMDS). 25 The NMDS is the national record of all public and private hospital discharge information, including coded clinical data for admissions greater than 4 hours for all of the New Zealand population. The recorded diagnoses are coded using the International Classification of Diseases and Related Health Problems Tenth Revision, Australian Modification (ICD-10-AM). 26 Patients were followed from their first dispensing of dabigatran etexilate until the date of hospitalization, cessation of dabigatran etexilate treatment, or study end.

**Table 1:** Wilcoxon's signed-ranks test and weighted kappa comparison of baseline renal function of the Cockcroft–Gault equation with the CKD-EPI and CKD-EPI-BSA equations for patients dispensed dabigatran etexilate

Age (y)	Cockcroft–Gault (median (IQR), mL/min)	CKD-EPI (median (IQR), mL/min/1.73 m ^2^ ); *p* -value	Weighted kappa; *p* -value	CKD-EPI-BSA (median (IQR), mL/min); *p* -value	Weighted kappa; *p* -value
<65 ( *n* = 564)	113.2 (92.6–144.1)	80.1 (69.7–92.5); <0.05	−0.01; <0.05	100.0 (85.7–119.2); <0.05	0.24; <0.05
65–74 ( *n* = 898)	80.6 (66.6–96.1)	69.1 (58.5–79.0); <0.05	0.41; <0.05	80.8 (68.2–94.9; <0.05)	0.64; <0.05
75–79 ( *n* = 452)	62.9 (51.5–76.1)	62.7 (51.2–73.7); <0.05	0.48; <0.05	68.9 (55.5–83.2); <0.05	0.75; <0.05
>80 ( *n* = 511)	50.0 (40.7–60.6)	57.0 (46.9–66.0); <0.05	0.39; <0.05	59.5 (49.2–70.6); <0.05	0.62; <0.05

Abbreviations: CKD-EPI, chronic kidney disease epidemiology initiative equation; CKD-EPI-BSA, chronic kidney disease epidemiology initiative equation adjusted for body surface area.

### Statistical Analyses

Statistical analyses were performed using Stata/IC (Version 14.2, StataCorpLP, Texas, United States). Continuous variables were tested for normal distribution by the skewness and kurtosis test. Normally distributed data are presented as the mean ± standard deviation and nonnormally distributed data as the median (interquartile range [IQR]) with between-group comparisons tested with paired-samples *t* -test. Categorical variables were expressed as percentages and compared by chi-square tests. Analysis of variance (ANOVA) and the Wilcoxon signed-ranks test were used to compare the difference in renal clearance in relation to age. Weighted Cohen's kappa coefficient of agreement was used as a measure of agreement between the equations. Differences in the number of patients categorized in the three dose stratifications based on CrCl (as calculated using Cockcroft–Gault) compared with eGFR (as calculated using either CKD-EPI or CKD-EPI-BSA) were compared using the chi-square Fisher's exact test. Discordance rates of dosing were calculated as the percentage of patients with a different dose than that determined by the Cockcroft–Gault equation divided by the total number of patients multiplied by 100. Hazard ratios (95% confidence interval [CI]) comparing different estimates of renal function and the actual dose of dabigatran etexilate dispensed (i.e., correct, low, or high doses) were derived from Cox's proportional hazard models. Results were considered statistically significant if *p* < 0.05.

## Results

### Patient Characteristics

There were 2,425 patients identified in the databases that had been dispensed dabigatran etexilate with a diagnosis of AF, aged 18 years or more, one or more serum creatinine, and bodyweight and height measurements recorded. The median age of patients in this cohort was 72 years (IQR: 65–78 years) and 1,417 (58.4%) were male. The median bodyweight was 85 kg (IQR: 73–100 kg), median height was 1.7 m (IQR: 1.6–1.8 m), and median BSA was 2.0 m ^2^ (IQR: 1.8–2.2 m ^2^ ).

### Baseline Renal Function Estimation

**Fig. 1:**
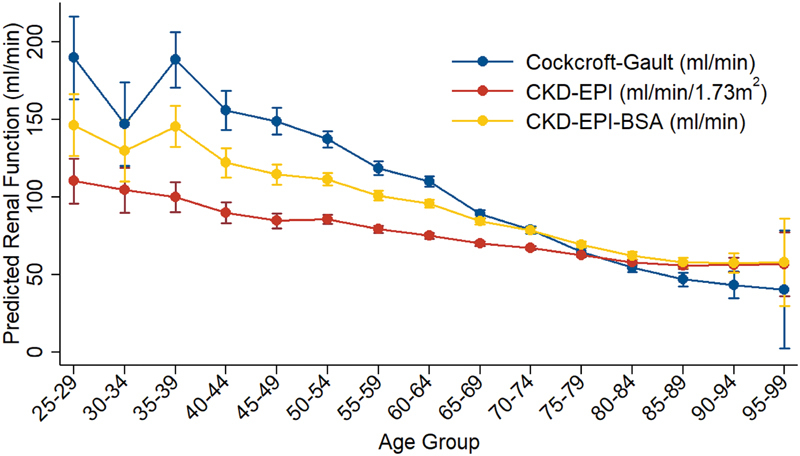
Adjusted predictions (ANOVA) of baseline renal function with 95% CI for patients dispensed dabigatran etexilate by the Cockcroft–Gault equation, CKD-EPI equation, and CKD-EPI-BSA equation ( *n* = 2,425).

Patient renal function decreased significantly ( *p* < 0.05) in relation to increasing age for the three different estimations ( Fig. 1 ). Comparing the level of agreement between the equations yielded a weighted kappa coefficient of 0.47 ( *p* < 0.05) between Cockcroft–Gault and CKD-EPI and 0.71 ( *p* < 0.05) between Cockcroft–Gault and CKD-EPI-BSA. Comparisons at the specified age stratifications showed similar levels of agreement with fair agreement for CKD-EPI and moderate agreement for CKD-EPI-BSA ( Table 1 ). When comparing the Cockcroft–Gault equation to the CKD-EPI and CKD-EPI-BSA equations across all age ranges, the Cockcroft–Gault equation produced a significantly higher estimated median renal function, 74.9 mL/min (IQR: 55.8–99.1 mL/min), than CKD-EPI, 67.6 mL/min/1.73 m ^2^ (IQR: 55.4–79.8 mL/min/1.73 m ^2^ ; *p* < 0.05). When comparing Cockcroft–Gault to the CKD-EPI equation at the specified age stratifications, CKD-EPI significantly underestimated until 80 years of age when it then significantly overestimated ( Table 1 ). When comparing Cockcroft–Gault to the CKD-EPI-BSA equation, the Cockcroft–Gault equation produced nonsignificant lower estimated median renal function than CKD-EPI-BSA, 77.4 mL/min (IQR: 60.7–95.4 mL/min; *p* = 0.968). When comparing Cockcroft–Gault to CKD-EPI-BSA equation over the specified age stratifications, CKD-EPI-BSA gave significantly lower estimates until 75 years of age when it then significantly overestimated ( Table 1 ).

**Fig. 2:**
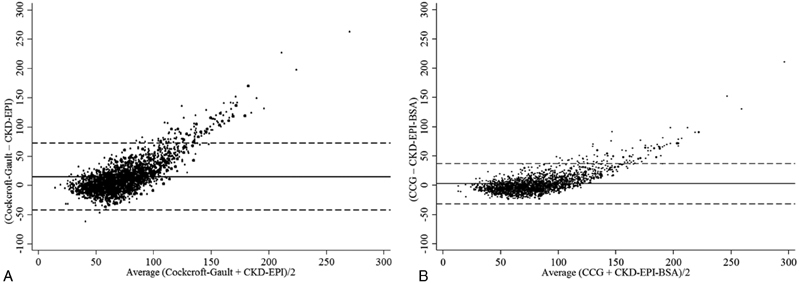
Bland and Altman plots showing the within-person differences between the estimated CrCl obtained by using the Cockcroft–Gault equation and eGFR obtained by using the CKD-EPI equation **(A)** and CKD-EPI adjusted for body surface area **(B)** . The *solid line* indicates the mean difference and the *dashed line* indicates limits of agreement. CKD-EPI, chronic disease epidemiology collaboration; CKD-EPI-BSA, chronic disease epidemiology collaboration adjusted for body surface area.

Both CKD-EPI and CKD-EPI-BSA provided biased estimates of renal function when compared with Cockcroft–Gault, with the bias being smaller for CKD-EPI-BSA ( Fig. 2 ). The mean within-patient differences relative to the Cockcroft–Gault equation were 14.8 mL/min (95% CI: 13.6–15.9) for CKD-EPI ( *p* < 0.05) and 2.6 mL/min (95% CI: 1.9–3.2) for CKD-EPI-BSA ( *p* < 0.05). The limits of agreement of CKD-EPI and CKD-EPI-BSA with the Cockcroft–Gault equation were −42.5 to 72.0 mL/min for CKD-EPI and −31.2 to 36.3 mL/min for CKD-EPI-BSA

Table 2 shows the renal dose stratification of patients according to the different renal function estimations. There were significant differences in all the renal dose stratifications of <30 mL/min and 30 to 49 mL/min produced by CKD-EPI and CKD-EPI-BSA compared with Cockcroft–Gault ( *p* < 0.05). For patients with renal dose stratifications of ≥50 mL/min, there was a significant difference for CKD-EPI compared with Cockcroft–Gault ( *p* < 0.05), while there was a nonsignificant difference for CKD-EPI-BSA compared with Cockcroft–Gault ( *p* = 0.241).

**Table 2:** Distribution of patients in each renal dose stratification level for dabigatran etexilate

CrCl/eGFR (mL/min)	% of patients in each dosing range			Cockcroft–Gault vs. CKD-EPI *p* -value	Cockcroft–Gault vs. CKD-EPI-BSA *p* -value
	Cockcroft–Gault (mL/min)	CKD-EPI (mL/min)/1.73 m ^2^	CKD-EPI-BSA (mL/min)		
<30	1.5	0.8	0.6	<0.05	<0.05
30–49	15.7	15.2	10.1	<0.05	<0.05
≥50	82.9	84.0	89.3	<0.05	<0.05

Abbreviations: CKD-EPI, chronic kidney disease epidemiology initiative equation; CKD-EPI-BSA, chronic kidney disease epidemiology initiative equation adjusted for body surface area; CrCl, creatinine clearance; eGFR, estimated glomerular filtration rate.

Discordance rates in the recommended doses determined using the CKD-EPI or CKD-EPI-BSA equations compared with Cockcroft–Gault are shown in Table 3 . For both estimators, disagreement in dose across all age ranges was because of an overall higher dose recommended by CKD-EPI and CKD-EPI-BSA compared with the Cockcroft–Gault dose. When discordance rates for dabigatran etexilate were evaluated by age grouping, the discordance rate increased with each increasing age with the most discordance occurring in patients 80 years and over ( Table 3 ).

**Table 3:** Percent dosing discordance for CKD-EPI and CKD-EPI-BSA vs. Cockcroft–Gault for dabigatran etexilate by age category

Age category	*n*	Cockcroft–Gault vs. CKD-EPI			Cockcroft–Gault vs. CKD-EPI-BSA		
		Discordance %	Underdose %	Overdose %	Discordance %	Underdose %	Overdose %
All patients	2,425	15.8	7.0	8.8	8.7	3.3	5.4
<65 y	564	5.8	5.3	0.5	2.0	1.8	0.2
65–74 y	898	9.7	7.1	2.6	5.3	3.7	1.6
75–79 y	452	20.0	10.0	10.0	9.3	4.0	5.3
≥80 y	511	33.9	6.1	27.8	21.7	3.9	17.8

Abbreviations: CKD-EPI, chronic kidney disease epidemiology initiative equation; CKD-EPI-BSA, chronic kidney disease epidemiology initiative equation adjusted for body surface area.

### Adverse Events Requiring Hospitalization

Approximately 9% of patients required hospitalization due to an adverse event possibly related to dabigatran etexilate; there were 138 (5.7%) hemorrhagic events, 45 (1.9%) thromboembolic/cerebrovascular accident (CVA) events and 33 (1.4%) unspecified CVA. The median follow-up for patients was 1.4 years (IQR: 0.4–2.5 years). For adverse events, the median time to a hemorrhagic event was 1.0 year (IQR: 0.3–1.9 years), cerebral vascular incident or systemic embolism was 0.9 years (IQR: 0.4–1.8 years), and an unspecified cerebral vascular incident was 0.9 years (IQR: 0.2–1.6 years).

While all methods showed that they were protective of a hemorrhage at the correct dose, only the Cockcroft–Gault estimation significantly indicated that those with a high dose dispensed were more likely to have a hemorrhage ( Table 4 ). Hence, if the Cockcroft–Gault equation is not used to guide dosing, there is an associated increased risk of hemorrhagic event. However, dosing by CKD-EPI or CKD-EPI-BSA did not provide guidance that would enable dose modification to prevent these events.

**Table 4:** Estimated hazard ratio (HR) and 95% confidence interval (95% CI) of hemorrhage, thromboembolism/CVA, or unspecified CVA, by actual dose dispensed compared with renal function estimator method

	Hemorrhage	Thromboembolism/CVA	Unspecified CVA
	HR (95%CI; *p* -value)	HR (95%CI; *p* -value)	HR (95%CI; *p* -value)
**Cockcroft–Gault**			
Correct dose ( *n* = 1,503)	** 0.57 a (0.41–0.79; 0.001) **	0.57 (0.32–1.02; 0.057)	0.71 (0.36–1.42; 0.335)
High dose ( *n* = 85)	** 2.32 a (1.22–4.42; 0.01) **	– b	0.93 (0.13–6.84; 0.947)
Low dose ( *n* = 837)	** 1.52 a (1.08–2.12; 0.015) **	** 2.04 a (1.13–3.65; 0.017) **	1.43 (0.72–2.85; 0.313)
**CKD-EPI**			
Correct dose ( *n* = 1,463)	** 0.66 a (0.47–0.92; 0.014) **	0.6 (0.34–1.08; 0.09)	0.67 (0.34–1.33; 0.251)
High dose ( *n* = 84)	1.06 (0.43–2.58; 0.904)	– b	– b
Low dose ( *n* = 878)	** 1.52 a (1.09–2.13; 0.014) **	** 1.92 a (1.07–3.44; 0.029) **	1.72 (0.87–3.42; 0.118)
**CKD-EPI-BSA**			
Correct dose ( *n* = 1,416)	** 0.66 a (0.48–0.93; 0.016) **	0.56 (0.31–1; 0.052)	0.51 (0.26–1.02; 0.058)
High dose ( *n* = 40)	1.17 (0.37–3.66; 0.792)	– b	– b
Low dose ( *n* = 969)	** 1.49 a (1.07–2.08; 0.019) **	** 1.94 a (1.08–3.5; 0.027) **	** 2.11 a (1.06–4.2; 0.035) **

Abbreviations: CKD-EPI, chronic kidney disease epidemiology initiative equation; CKD-EPI-BSA, chronic kidney disease epidemiology initiative equation adjusted for body surface area; CVA, cerebrovascular accident; HR, hazard ratio.

a Statistically significant.

b Not included in the model due to insufficient numbers.

## Discussion

This study demonstrates that substituting renal function estimated by CrCl (Cockcroft–Gault) with eGFR (CKD-EPI or CKD-EPI-BSA) can also negatively influence adverse outcomes with dabigatran etexilate for some patients, with elderly patients having the highest risk. This is likely to be the result of higher doses of dabigatran etexilate being prescribed for patients whose renal function is near the limits of the recommended dose stratifications.

Using individual patient's data, we determined doses of dabigatran etexilate if the prescriber used the different methods to estimate renal function. We then compared the difference in the dose they would receive based on the CKD-EPI and CKD-EPI-BSA equations with the Cockcroft–Gault equation, as this is the method used for dose stratification in the medicines summary of product characteristics, to determine dose discordance. The greatest dose discordance compared with the Cockcroft–Gault equation was observed for: (1) patients over 75 years of age for CKD-EPI (received a supratherapeutic dose) and (2) patients over 80 years of age for CKD-EPI-BSA (received a supratherapeutic dose).

Hence, it appears the choice of renal function estimator is important for individuals over 75 years of age. It is at these points that patients with a CrCl below the recommended dosing stratifications (i.e., 50 and 30 mL/min) are likely to be estimated higher with eGFR and therefore receive a clinically inappropriate high dose. This indicates that the CKD-EPI and CKD-EPI-BSA equations should not be used as an alternative for Cockcroft–Gault equation when estimating renal function to guide dabigatran etexilate dosing in AF. This is especially important for elderly patients as renal function is known to decline more rapidly in patients with AF. 27

While previous studies have demonstrated, through simulations, that there would be clinically important risks to prescribing practice for dabigatran etexilate if alternatives to the Cockcroft–Gault equation were used to estimate renal function, 1 2 3 4 none have investigated this with patient outcomes data. This study is the first, to our knowledge, to examine a large cohort of patients dispensed dabigatran etexilate and investigate the impact of different estimates of renal function on adverse outcomes.

Of the methods investigated, only high doses of dabigatran etexilate estimated by the Cockcroft–Gault equation showed a statistically significant increased hazard ratio of 2.32 (95% CI: 1.22–4.42; *p* = 0.001) for a hemorrhage. The present study indicates that the Cockcroft–Gault equation is a better predictor of appropriate dabigatran etexilate dosing than the CKD-EPI and CKD-EPI-BSA equations as they do not effectively prevent the recommendation of inappropriately high doses of dabigatran. This can be attributed to those patients whose renal function is nearing the recommended dose stratification limits and being overestimated by the CKD-EPI and CKD-EPI-BSA equations—thus, not receiving a clinically appropriate dose reduction based on renal function and suffering a hemorrhage. Additionally, the equations CKD-EPI and CKD-EPI-BSA showed only fair to moderate agreement (weighted kappa coefficients of 0.47 and 0.71, respectively) with the Cockcroft–Gault equation for this cohort of patients. This indicates that the Cockcroft–Gault equation should not be substituted by the CKD-EPI and CKD-EPI-BSA equations for estimating renal function when determining the dose of dabigatran etexilate, especially in the elderly.

These findings are important, especially for elderly patients, as rates of bleeding and CVA increase as renal function deteriorates, 27 28 and declines in renal function are known to occur with aging. 29 Additionally, a previous study has reported that elderly patients, receiving dabigatran etexilate, were at a greater risk of major gastrointestinal hemorrhagic bleeding compared with warfarin. 30 This makes it imperative that a clinically appropriate dose of dabigatran etexilate is selected and hence the Cockcroft–Gault estimator of renal function is used.

The limitations of this study include the NMDS only capturing patient data for those who require in-patient hospitalization for a duration of more than 4 hours. Therefore, any outcomes of interest that did not meet these criteria, for example, a hemorrhage or CVA that resulted in death without an in-patient hospitalization, would not be included in the dataset, resulting in possible underestimations. Additionally, there is the possibility of errors in the clinical information from the NMDS and primary care dataset. These errors could result in inclusion or exclusion of clinical outcomes of interest. However, it has been reported that there is high sensitivity when using ICD-9-CM to identify hemorrhagic events with 93% sensitivity and 88% specificity to identifying a definite major hemorrhagic event. 31 Similarly, it has been reported that using ICD-10 to identify CVA has a positive predictive value (PPV) of close to or greater than 90% and therefore adequate to identify CVA. 32 We assumed that the ICD-10-AM used to identify hemorrhagic events had high sensitivity and specificity and those used to identify CVA had a high PPV. These limitations contribute to background variability but would not be expected to contribute to a systematic bias. There was no access to reliable information about patient comorbidities, and as their interaction with dabigatran etexilate treatment is not accounted for in this study, there is possible bias in outcome profiles. For example, it was not possible to determine the individual patient CHA _2_ DS _2_ -VASc and HAS-BLED scores. These assessments quantify thromboembolic risk versus bleeding risk and can result in dose recommendations that differ from those derived solely from renal function. With no diagnostic information contained in the NMDS, it was therefore not possible to investigate disease-specific dose regimens by indication. These limitations would all be expected to contribute to random variability, but would not be expected to result in any systematic bias. Additionally, the PC only provides information relating to the dispensing of medications and it is not possible to confirm if the patients within this cohort have adhered to the prescribed regimen. The MDRD-4 equation was not analyzed in this study as this test is now infrequently used in New Zealand with CKD-EPI the estimate of choice for laboratory reporting. Therefore, the MDRD-4 equation was unlikely to have been utilized by clinicians to determine dosing and thus its relative performance has not been investigated. Also, receiver operating characteristic plot analysis did not indicate that the different renal function estimations were reliable predictors of outcomes. This might be due to dose modifications that may have been made based on the test result or patient covariates, which would obscure the relationship. The main strength of this study is the inclusion of a large cohort of patients with a similar age profile to that of the original clinical trial, with sufficient sample size to provide adequate information about the effects of different renal function estimators on dabigatran etexilate outcomes for an entire population.

The results of the present study indicate that when determining dabigatran etexilate dose adjustments for renal function, clinicians should use the method utilized in the clinical trial pharmacokinetic studies (i.e., the Cockcroft–Gault equation). This is particularly pertinent in primary care as dabigatran prescribing is increasingly used in this setting. 4 33 Although there is familiarity with estimating renal function using the Cockcroft–Gault equation in hospital practice, this is not the case in primary care. 4 Additionally, it has been previously reported that clinicians predominately use eGFR reported by laboratories 16 ; therefore, alternate methods of reporting renal function estimated via the Cockcroft–Gault equation need to be investigated. This could be in the form of automated calculators integrated within the clinician's EHR. Furthermore, with it being reported that CKD-EPI gives the best estimation of glomerular filtration rate, 12 it may be prudent that regulatory bodies, such as the FDA and EMA, consider mandatory use of this method for the evaluation of medicines in patients with decreased renal function.

## Conclusion

Both CKD-EPI and CKD-EPI-BSA equations significantly overestimated renal function in the elderly population compared with the Cockcroft–Gault equation. This may lead to dose selection errors for dabigatran etexilate, particularly for those with severe impairment, increasing the risk of an adverse outcome. Hence, CKD-EPI and CKD-EPI-BSA equations should not be substituted in place of the Cockcroft–Gault equation in older adults for the purpose of renal dosage adjustments.
