# Supplementary material for: Choice of Renal Function Estimator Influences Adverse Outcomes with Dabigatran Etexilate in Patients with Atrial Fibrillation
Source: TH Open. 2018 Dec 10;2(4):e420–7. doi: 10.1055/s-0038-1676356 (PMC6524914; doi:10.1055/s-0038-1676356)
Supplement: Supplementary file 1 — Supplementary Material [file 10-1055-s-0038-1676356-s180045.pdf]

Supplementary Table 1 ICD-10-AM codes used for the identification of hemorrhage, thromboembolism/CVA, and unspecified CVA

Adverse event type	ICD-10-AM code
Hemorrhage	**D683** (Hemorrhagic disorder due to extrinsic circulating anticoagulants), **H431** (Vitreous hemorrhage), **I601** (Nontraumatic subarachnoid hemorrhage from middle cerebral artery), **I609** (Nontraumatic subarachnoid hemorrhage, unspecified), **I610** (Nontraumatic intracerebral hemorrhage in hemisphere, subcortical), **I611** (Nontraumatic intracerebral hemorrhage in hemisphere, cortical), **I613** (Nontraumatic intracerebral hemorrhage in brain stem), **I615** (Nontraumatic intracerebral hemorrhage, intraventricular), **I616** (Nontraumatic intracerebral hemorrhage, multiple localized), **I618** (Other nontraumatic intracerebral hemorrhage), **I619** (Nontraumatic intracerebral hemorrhage, unspecified), **I620** (Nontraumatic subdural hemorrhage), **I629** (Nontraumatic intracranial hemorrhage, unspecified), **I671** (Cerebral aneurysm, nonruptured), **K250** (Acute gastric ulcer with hemorrhage), **K254** (Chronic or unspecified gastric ulcer with hemorrhage), **K260** (Acute duodenal ulcer with hemorrhage), **K264** (Chronic or unspecified duodenal ulcer with hemorrhage), **K266** (Chronic or unspecified duodenal ulcer with both hemorrhage and perforation), **K274** (Chronic or unspecified peptic ulcer, site unspecified, with hemorrhage), **K284** (Chronic or unspecified gastrojejunal ulcer with hemorrhage), **K290** (Acute gastritis with bleeding), **K625** (Hemorrhage of anus and rectum), **K661** (Hemoperitoneum), **K920** (Hematemesis), **K921** (Melena), **K922** (Gastrointestinal hemorrhage, unspecified), **N028** (Recurrent and persistent hematuria with other morphologic changes), **N029** (Recurrent and persistent hematuria with unspecified morphologic changes), **N938** (Other specified abnormal uterine and vaginal bleeding), **N939** (Abnormal uterine and vaginal bleeding, unspecified), **N950** (Postmenopausal bleeding), R042 (Hemoptysis), **R048** (Hemorrhage from other sites in respiratory passages), **R58** (Hemorrhage, not elsewhere classified)
Thromboembolism/ CVA	**I634** (Cerebral infarction due to embolism of other cerebral artery), **I631** (Cerebral infarction due to embolism of precerebral arteries), **I633** (Cerebral infarction due to thrombosis of cerebral arteries), **I630** (Cerebral infarction due to thrombosis of precerebral arteries), **I632** (Cerebral infarction due to unspecified occlusion or stenosis of precerebral arteries), **I639** (Cerebral infarction, unspecified), **I744** (Embolism and thrombosis of arteries of extremities, unspecified), **I743** (Embolism and thrombosis of arteries of the lower extremities), **I742** (Embolism and thrombosis of arteries of the upper extremities), **I652** (Occlusion and stenosis of carotid artery), **I663** (Occlusion and stenosis of cerebellar arteries), **I660** (Occlusion and stenosis of middle cerebral artery), **I653** (Occlusion and stenosis of precerebral arteries, not resulting in cerebral infarction), **I668** (Occlusion and stenosis of other cerebral arteries), **I658** (Occlusion and stenosis of other precerebral arteries), I669 (Occlusion and stenosis of unspecified cerebral artery), **I659** (Occlusion and stenosis of unspecified precerebral artery), **G458** (Other transient cerebral ischemic attacks and related syndromes), **I64** (Other transient cerebral ischemic attacks and related syndromes), **G459** (Transient cerebral ischemic attack, unspecified), **G450** (Vertebrobasilar artery syndrome)
Unspecified CVA	**I635** (Cerebral infarction due to unspecified occlusion or stenosis of cerebral arteries), **I679** (Cerebrovascular disease, unspecified), **I678** (Other specified cerebrovascular diseases), **I638** (Other cerebral infarction)
